# Taguchi L9 Optimization of Microwave‐Assisted Solid–Liquid Duo Heating for Berberine Extraction With HPTLC Analysis

**DOI:** 10.1002/bmc.70008

**Published:** 2025-02-07

**Authors:** Saurabh Satija, Manish Vyas, Parijat Pandey, Munish Garg

**Affiliations:** ^1^ School of Medical Indigenous and Health Sciences, University of Wollongong Wollongong New South Wales Australia; ^2^ Molecular Horizons, Faculty of Science, Medicine & Health University of Wollongong Wollongong New South Wales Australia; ^3^ School of Pharmaceutical Sciences Lovely Professional University Phagwara India; ^4^ Department of Pharmaceutical Sciences Gurugram University Gurugram India; ^5^ Department of Pharmaceutical Sciences Maharshi Dayanand University Rohtak India

**Keywords:** berberine, HPTLC, microwave‐assisted extraction, Taguchi

## Abstract

We present an optimized approach for the extraction of berberine using microwave‐assisted solid–liquid duo heating. The conventional procedure relies on the simultaneous heating of the sample matrix and the dissolving solvent under microwave energy. To improve the efficiency of this process, we employed the Taguchi L9 orthogonal design methodology, focusing on key extraction parameters, including microwave power, irradiation time, solute‐solvent ratio, and temperature. Berberine content was quantified using high‐performance thin‐layer chromatography (HPTLC). Our findings revealed that the most influential parameters in the berberine extraction process were microwave power (ranked as the most critical factor), followed by irradiation time (second in importance), and temperature (third in significance). Utilizing the dual heating approach in microwave‐assisted extraction (MAE) led to a more precise and efficient determination of extraction parameters, resulting in enhanced accuracy and higher yield. Additionally, this optimized method significantly reduced the overall extraction time, making it an ideal choice for berberine extraction under optimal conditions.

## 1 Introduction

Microwave‐assisted extraction (MAE) utilizes microwave energy to heat solvents that interact directly with a sample, promoting the transfer of analytes from the sample matrix into the solvent. The rapid heating characteristic of MAE provides a distinct advantage by allowing swift temperature elevation of the sample‐solvent system. Sealed vessel operation enables extraction at higher temperatures, which accelerates the movement of targeted compounds from the sample matrix (Satija et al. 2015). This process involves a phenomenon where cells rupture due to localized superheating, releasing active constituents. This rupture may facilitate solvent access to dissolve the target analyte more effectively, enhancing extraction speed and efficiency. Compared with traditional methods such as soxhlet and maceration, MAE reduces both extraction time and solvent requirements, offering increased extraction rates and some selectivity. Numerous studies have applied MAE to extract bioactive compounds (Fulzele and Satdive 2005). Unlike conventional Soxhlet extraction, MAE heating relies on the dielectric properties of the solvent and matrix in open‐vessel systems (Satija et al. 2015), which produces distinct heating dynamics. Various heating mechanisms specific to MAE have been identified (Karimi and Jaafar 2011).

A Taguchi design is employed to select a product or process that functions more consistently within its operational environment. It acknowledges that not all factors contributing to variability can be controlled, labeling these uncontrollable factors as noise factors. Taguchi designs aim to identify controllable elements, referred to as control factors, that minimize the impact of these noise factors. During experimentation, noise factors are intentionally manipulated to induce variability, allowing for the determination of optimal control factor settings that render the process or product robust, or resistant to variations caused by the noise factors. A process engineered with the objective of screening test factors will yield more consistent outputs, and a product designed with this goal will exhibit more consistent performance regardless of the environmental conditions it encounters (Yang 2001). Taguchi designs employ symmetrical arrays to assess the effects of factors on response mean and variability. Symmetrical arrays ensure that the design is balanced, giving equal weight to factor levels. Consequently, each factor can be evaluated independently of the others, preventing one factor's influence from affecting the assessment of another. This approach can significantly reduce the time and cost associated with research when fractional designs are utilized, with a primary focus on main effects (Karimi and Jaafar 2011).

Berberine, an alkaloid extracted from the stem of *Tinospora cordifolia* (Satija, Malik, and Garg 2016), is extensively used in Ayurvedic medicine for its wide‐ranging therapeutic effects, including anticancer, antiulcer, antipyretic, antihepatitis, immunomodulatory, antioxidant, and hypoglycemic properties (Pradhan, Ojha, and Pandey 2013; Manikyam et al. 2021). As a major active compound in *Tinospora cordifolia*, berberine is responsible for many of the plant's medicinal benefits. This paper introduces a new MAE method designed for the rapid and efficient extraction of berberine, using a system that simultaneously heats both the solvent and the powdered plant material. Optimization of the extraction process was achieved through a Taguchi L9 orthogonal design, allowing for the identification of key parameters such as microwave power, irradiation time, solute‐to‐solvent ratio, and temperature.

## 2 Experimental

### 2.1 Instrumental

MAE was performed using a UWave‐1000 Microwave‐Ultraviolet‐Ultrasonic Synthesis Extraction Reactor (Sineo Microwave Chemistry Technology Co. Ltd., China). This system has a mono‐mode operation and can reach a maximum power of 1000 W, with settings to control both temperature and power over time. A platinum probe positioned inside the microwave cavity was used for temperature monitoring throughout the process. The concentration of berberine in the extracts was analyzed on a Camag HPTLC system, which included components such as a Camag Linomat 5 automatic sampler, TLC Plate Heater III for plate drying, a developing chamber for plate development, a TLC Visualizer for image capture, and a TLC Scanner 3 for plate scanning. Sample injections were done using a Camag Linomat syringe (695.0014, Hamilton Bonaduz, Schweiz).

### 2.2 Experimental Design

The Taguchi‐based optimization approach was customized to improve the MAE of *Tinospora cordifolia* using a dual heating system. In this process, four key parameters—microwave power (A), irradiation time (B), solute‐to‐solvent ratio (C), and temperature (D)—were initially characterized, as presented in Table 1. The percentage berberine content, labeled as response variable Y, was measured for analysis. An L9 orthogonal array was utilized to streamline the optimization, necessitating nine experimental trials as summarized in Table 2. Data from these trials were analyzed using Minitab software, version 17.3. To prevent any potential personal or subjective bias, the sequence of experiments was randomized.

**TABLE 1:** Factors and levels for the orthogonal design (A–D are the respective codes for each factor).

	Microwave power (W)	Irradiation time (min)	Solute:solvent ratio (w/v; g/mL)	Temperature (°C)
Levels	A	B	C	D
1	300	1	1:10	50
2	400	2	1:15	60
3	500	3	1:20	70

**TABLE 2:** Experimental design and results of L_9_ (3^4^) orthogonal array.

Batch no.	Factors				Response
	Microwave power (W) A	Irradiation time (min) B	Solute:solvent ratio (w/v; g/mL) C	Temperature (°C) D	Percentage berberine content (w/w) mean of response Ӯ
T1	1 (300)^a^	1 (1)	1 (1:10)	1 (50)	1.198
T2	1 (300)	2 (2)	2 (1:15)	2 (60)	1.583
T 3	1 (300)	3 (3)	3 (1:20)	3 (70)	2.179
T 4	2 (400)	1 (1)	2 (1:15)	3 (70)	2.018
T 5	2 (400)	2 (2)	3 (1:20)	1 (50)	1.731
T 6	2 (400)	3 (3)	1 (1:10)	2 (60)	1.981
T 7	3 (500)	1 (1)	3 (1:20)	2 (60)	2.589
T 8	3 (500)	2 (2)	1 (1:10)	3 (70)	2.973
V9	3 (500)	3 (3)	2 (1:15)	1 (50)	3.000

*Note:* Ӯ represents mean of response; *n* = 3.

^a^ The value in the bracket indicates the real values.

### 2.3 Solid–Liquid Duo Heating Mechanism Based MAE

In the MAE procedure, the accurately weighed drug powder was thoroughly combined with methanol, the chosen extraction solvent, following the specifications of the experimental design. The prepared powder was then placed into the extraction vessel, and the required volume of solvent was added. Extraction was carried out under varying microwave power levels and irradiation times, as set by the experimental protocol. After extraction, the mixture was filtered, concentrated under vacuum, and redissolved in methanol for quantification via HPTLC.

### 2.4 Quantification by HPTLC

Berberine in *Tinospora cordifolia* extracts was quantified using a HPTLC method based on established protocols (Satija et al. 2020; Satija, Malik, and Garg 2016). For this analysis, 10 mg of each extract was added to a 10 mL volumetric flask and diluted with methanol to a final concentration of 100 μg/mL. A standard berberine solution was prepared by dissolving 10 mg of berberine in a 10 mL flask, then diluting to achieve a 10 μg/mL stock solution. For HPTLC, aliquots of 2, 3, 4, 5, 6, and 7 μL from this berberine stock (10 μg/mL) were applied to a 10 × 10 cm HPTLC plate, producing concentrations from 200 to 700 ng per spot. The microwave extracts were similarly screened by applying 4 μL of each test sample stock solution onto a separate TLC plate.

Samples were applied as 4 mm wide bands with a 12 mm distance between spots, using the CAMAG Linomat V applicator. Plate development was conducted in a pre‐saturated chamber with a mobile phase of methanol:acetic acid:water (8:1:1, v/v) for 20 min in an ascending mode, covering up to 80% of the plate length, using a CAMAG horizontal twin trough glass chamber at room temperature. After development, the plates were dried at 120 °C for 5 min on a CAMAG TLC plate heater, then visualized under UV and fluorescence reflectance mode at 366 nm using a CAMAG TLC visualizer system. A calibration curve was generated by plotting peak area against berberine concentration, enabling the quantification of berberine in sample spots. Berberine identity was confirmed by matching UV spectra within the same Rf range for both test and standard samples.

## 3 Results and Discussion

### 3.1 Impact of Solid–Liquid duo Heating MAE

Taguchi design was employed to investigate the relative importance of variables, including microwave power, irradiation time, solute‐solvent ratio, and temperature. A total of nine experiments (designated as batches T1–T9) were conducted within the framework of the Taguchi design. The berberine content in *Tinospora cordifolia* extracts was determined to be 2.49% (w/w) at an Rf value of 0.71. The extraction outcomes for batches T1–T9, carried out under orthogonal design conditions, are detailed in Table 2. All results presented at each stage of the design represent the mean values of three independent experiments. The ranking of these factors (as depicted in Table 3) was determined using Minitab 17 software, with the SN (signal‐to‐noise) value of each factor set to the default “Nominal is the best” in accordance with the software's specifications.

$$S/N=10\times \left(\left({\bar{y}}^{2}-s^{2}/n\right)/s^{2}\right)$$

where Ӯ = mean of response; s = sum of values; n = number of trial experiments.

**TABLE 3:** Ranking of factors.

Levels	A	B	C	D
1	1.653	1.935	2.051	1.976
2	1.910	2.096	2.200	2.051
3	2.854	2.387	2.166	2.390
Delta (Δ)	1.201	0.452	0.150	0.414
Rank	1	2	4	3

The selection of the “Nominal is Best” signal‐to‐noise ratio as the standard option is attributed to its effectiveness in evaluating scaling factors, where both the mean and standard deviation change proportionally. Scaling factors are used to adjust the mean towards a specified target without influencing the signal‐to‐noise ratios. The term “Delta” (Δ) indicates the total change in value, representing the difference between the highest and lowest mean response across various levels of a factor.

Within the Taguchi design methodology, the evaluation identified microwave power (Rank 1 ‐ A) as the most significant factor affecting the MAE of berberine, followed by irradiation time (Rank 2 ‐ B) and temperature (Rank 3 ‐ C). In contrast, the solute‐solvent ratio had a minimal impact on the extraction response and can thus be excluded from further response surface experiments. The Model *F*‐value of 11.19 indicates the statistical significance of the model, with only a 0.94% chance that such an *F*‐value could arise from random fluctuations.

### 3.2 Effect of Different Factors During MAE Extraction of Berberine From *Tinospora cordifolia*

**FIGURE 1:**
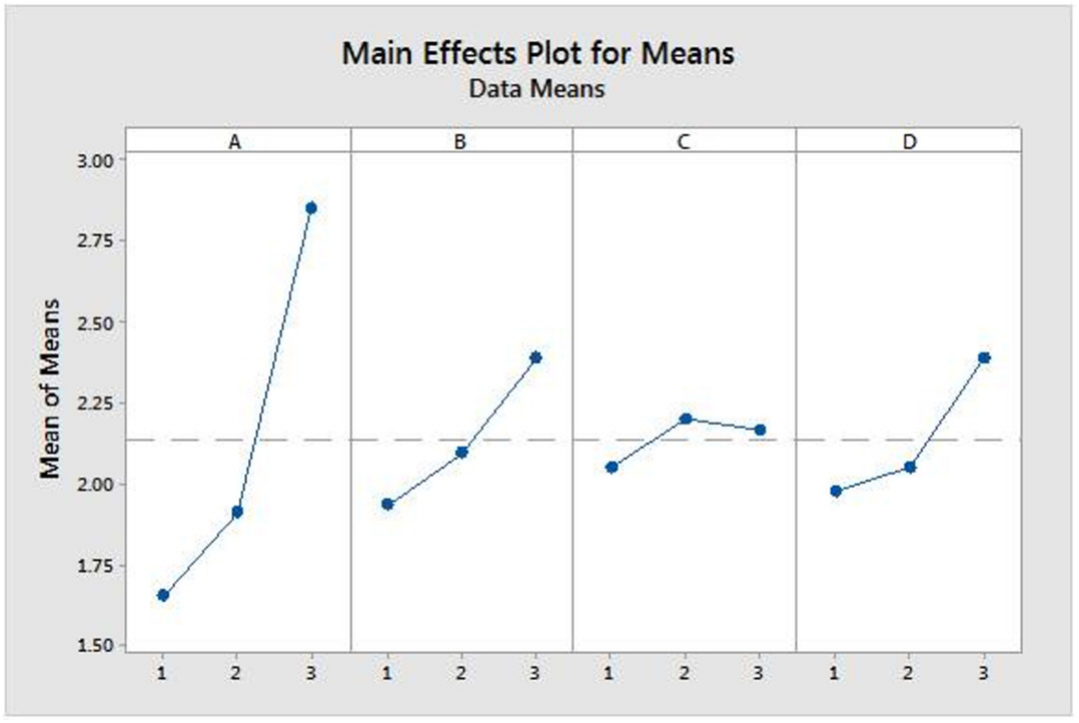
Main effect plot for means of different parameters at each level.

Given the numerous parameters involved in MAE, it becomes imperative to consider the influence of these parameters on the extraction process when optimizing conditions. To address this, we constructed a composite plot illustrating the primary effects of the methods, as depicted in Figure 1. The mean values depicted in this plot represent the average response for each combination of control factor levels.

The MAE strategy developed in this study serves as the most suitable screening model for optimizing parameters. It effectively identifies the most influential factors while allowing less critical elements to be disregarded, enhancing the precision of experimental design. This approach not only reduces the time and costs associated with research, especially when fractional designs are employed, but also serves as a valuable reference point for future factorial investigations in the context of MAE of medically significant bioactive compounds and their pharmaceutical formulations.

### 3.3 Applicability of the Proposed Method

The proposed MAE method is highly versatile and can potentially be extended to other phytochemicals with similar physicochemical properties as berberine. Compounds such as alkaloids, flavonoids, and phenolic acids, commonly found in medicinal plants, often exhibit sensitivity to heat and solvent polarity, making them suitable candidates for MAE. For instance, studies have demonstrated the effectiveness of MAE in the extraction of bioactive compounds from figs ( *Ficus racemosa* ) (Sharma et al. 2020). This adaptability is attributed to the uniform heating and reduced extraction time provided by microwave irradiation, which preserves the integrity of thermolabile compounds while enhancing yield.

### 3.4 Merits and Demerits

MAE offers several advantages over conventional extraction techniques like Soxhlet extraction, maceration, and ultrasonic‐assisted extraction (UAE). The primary merits include reduced extraction time, lower solvent consumption, and higher efficiency in releasing target compounds due to the rapid heating mechanism. Additionally, MAE minimizes thermal degradation of sensitive compounds, enhancing the quality of the extracted product.

However, certain limitations exist for example, the initial setup cost for MAE equipment can be higher compared to conventional systems, and the method's effectiveness may vary depending on the sample matrix and compound properties. Furthermore, scaling up for industrial applications requires optimization of parameters like solvent type, microwave power, and exposure time to ensure consistent results. Despite these challenges, MAE remains a promising technique for the extraction of bioactive compounds, offering a balance between efficiency and environmental sustainability.

## 4 Conclusion

This study introduces a novel MAE method for berberine extraction from *Tinospora cordifolia*, optimized through the Taguchi design framework. By employing this systematic approach, we efficiently evaluated multiple extraction parameters simultaneously, significantly enhancing extraction efficiency. The use of an L9 orthogonal array minimized the number of experimental runs required, saving both time and resources while ensuring robust statistical analysis. As a result of this optimization, we achieved increased berberine yield, demonstrating the effectiveness of the Taguchi method in maximizing the recovery of bioactive compounds. This research exemplifies the successful application of Taguchi optimization in phytochemical extraction, paving the way for more efficient methodologies in natural product development.

## Conflicts of Interest

The authors declare no conflicts of interest.
